# Molecular Detection of *Plasmodium falciparum* Infection in Matched Peripheral and Placental Blood Samples from Delivering Women in Libreville, Gabon

**DOI:** 10.1155/2014/486042

**Published:** 2014-11-17

**Authors:** Marie L. Tshibola Mbuyi, Marielle K. Bouyou-Akotet, Denise P. Mawili-Mboumba

**Affiliations:** Département de Parasitologie-Mycologie, Université des Sciences de la Santé, 4009 Libreville, Gabon

## Abstract

Submicroscopic infections account for more than 50% of all* Plasmodium (P.)* infections in areas with decreasing malaria prevalence and might contribute to poor pregnancy outcomes. The frequency of submicroscopic *P. falciparum* infections was assessed in matched peripheral and placental blood samples with microscopy negative or discordant results according to IPTp administration.* Methods*.* P. falciparum* infection was detected by nested PCR in matched blood samples collected from delivering women with a history of antimalarial drug treatment and living in Gabon.* Results*. Submicroscopic* P. falciparum* infections were detected in 87% (*n* = 33) of the 44 selected matched samples. Plasmodial DNA was found in 90% (*n* = 35/39) and 87% (*n* = 33/38) of microscopy negative peripheral and placental blood samples, respectively. Overall, 95% of samples obtained during the high IPTp-SP coverage period had a submicroscopic infection versus 79% among those from the low coverage period.* Conclusion*. Submicroscopic infections frequency is high in peripheral and placental blood samples from delivering women with a history of antimalarial treatment whatever the level of IPTp coverage. These data highlight the need of accurate diagnostic tools for a regular antenatal screening of malaria during the pregnancy in endemic areas.

## 1. Introduction

In 2010, more than 30 million of pregnant women in sub-Saharan Africa were exposed to malaria; among them 11.4 million were infected [1]. Malaria caused by* Plasmodium (P.) falciparum* has adverse effects on pregnancy and about a quarter of pregnant women present placental infection at delivery [2, 3]. Low birth weight is associated with neonatal morbidity and mortality [4]. This phenomenon occurs when malaria parasites are sequestered in the placenta, causing functional disorders of placental villosity and disrupting the fetomaternal compartment [5]. The World Health Organization (WHO) recommendations for malaria prevention during pregnancy were implemented in Gabon in 2005. Studies conducted in 2005 and 2007 at Libreville and Lambaréné confirmed the positive impact of intermittent preventive treatment during pregnancy with sulfadoxine-pyrimethamine (IPTp-SP) associated with the use of insecticide-treated nets (ITNs) on the rates of microscopy positive infections at delivery [6–8]. During the same period, most women with* P. falciparum *infections at delivery reported a history of fever treated with an antimalarial drug (HFTA), mostly on a presumptive basis [7]. As a single infection during pregnancy might contribute to poor outcome, it is important to monitor malaria throughout the entire period of gestation [9]. HFTA is a strong predictor of microscopic and submicroscopic infection at delivery [10, 11]. Indeed, parasites infecting pregnant women persist even after delivery, mostly as submicroscopic infections not detected on light microscopy examinations [10]. Recent reports have indicated that, in areas in which the prevalence of malaria has decreased, submicroscopic infections account for 70 to 80% of all* Plasmodium* infections in children, non pregnant adults, and pregnant women [12–15]. These submicroscopic infections are associated with adverse pregnancy outcomes [8, 10]. Thus, the aim of this study was to estimate the frequency of submicroscopic* P. falciparum* infections in matched peripheral and placental blood samples with microscopy negative or discordant results, and its relationship with IPTp administration.

## 2. Patients and Methods

### 2.1. Study Area

This study was conducted at the delivery unit of the* Centre Hospitalier Universitaire de Libreville *(CHUL; Libreville University Hospital), the largest public hospital in Libreville, the capital of Gabon. Malaria transmission is perennial in this area, with an estimated prevalence of 24%, and maternal microscopic peripheral infection at 6% [16, 17].* P. falciparum* is the most common parasite species identified [18]. The CHUL is a sentinel site for malaria surveillance, at which all pregnant women consulting for antenatal care (ANC) at public health centers have free access to a delivery unit.

### 2.2. Study Design and Sampling

Samples from delivering women who participated in two cross-sectional surveys for the estimation of malaria burden and outcomes during pregnancy at the time and after the implementation of IPTp were analyzed. Details on patient demographics and history of pregnancy are described elsewhere [16]. Each woman attending the delivery unit was interviewed by a field worker and the midwives for 15 minutes. Antenatal data were obtained from either the antenatal card or the register of the centre with assistance of the midwife; they included parity, gestational age at the interview, and use of malaria preventive strategies. If prevention was performed, the type of medicine taken was recorded as well as the first time of dosing, the number and dates of drug administration, and the number of tablets taken. Peripheral blood and placental blood obtained for the cleaned maternal face of the placenta (two mL) were collected from each woman for thick smears and molecular assays. The samples were selected for this study on the basis of two major criteria: having a HFTA during pregnancy and having peripheral and placental blood samples that gave concordant negative or discordant results after thick blood smear examinations.

### 2.3. Diagnosis of* P. falciparum* Infections

Matched thick and thin blood smears were prepared and stained with 20% Giemsa. Thick smears were screened for the presence of malaria parasites according to the Lambaréné method [19]. Carefully, 10 *μ*L of blood was laid on a 10 by 18 mm area of a microscope slide, then dried, and stained. The parasitemia was expressed as number of parasites per microliter of blood (p/*μ*L), and parasite species were identified in the matched thin blood smears. Smears were read by two experienced technicians using a light microscope (×100 oil immersion lenses). Smears were considered negative if no parasite was seen after the examination of at least 100 oil immersion fields in a thick blood smear. The definition of a malaria case was a febrile patient with a positive blood smear (PBS).

The blood smears were read by two experienced microscopists and in case of discordant results (presence or lack of asexual/sexual blood stages, mismatch species, or parasite density), the slides were reviewed by a third technician who resolved any discrepancy. For parasite density determination, the mean of the two closest parasitaemia was taken.

### 2.4. Molecular Detection of* P. falciparum* Infections

The blood samples were centrifuged to separate the pellet containing erythrocytes from the plasma, both of which were frozen at −80°C. DNA was extracted from the stored samples with the QIAamp DNA Mini Kit (Qiagen, Germany), according to the manufacturer's instructions. Five microliters of the extracted DNA were used as a template for* P. falciparum *detection. The merozoite surface protein 1* (msp1)* and merozoite surface protein 2* (msp2)* genes were amplified by nested PCR, as described elsewhere, on a Labnet Multigene II Thermal cycler [20]. Primary amplifications were run with primer pairs corresponding to the flanking sequence of the conserved regions of the* msp1* and* msp2* genes. The second amplification reactions were carried out with allele-specific primer sets corresponding to the* msp1 *(K1, RO33, and Mad20) and* msp2 *(FC27 and 3D7) allele families. A negative control sample without DNA template was used in all the reactions. PCR products were either stored at +4°C or analyzed immediately by electrophoresis in a 2.5% agarose gel.

### 2.5. Definitions

The matched samples were the peripheral and placental blood samples collected from the same woman. They were classified as concordant or discordant. Concordant samples were matched samples that gave the same results after microscopy examination of thick blood smears: positive (concordant positive) or negative (concordant negative). Discordant samples were paired peripheral and placental blood samples that gave different results after microscopy examination of thick blood smears: positive for the peripheral sample and negative for the placental sample, or vice versa. Samples with* P. falciparum* submicroscopic infection are those without detected parasites on thick and thin blood smear (microscopy negative) but with a positive PCR result (i.e., detected plasmodial DNA). IPTp-SP coverage was defined as the proportion of women taking at least one dose of SP during pregnancy. Thus, 2005, a year in which close to 35% of pregnant women received a single dose of SP, was classified as a period of low IPTp-SP coverage. By contrast, 2011, a year in which more than 80% of pregnant women took at least one single dose of SP, was classified as a period of high IPTp-SP coverage.

### 2.6. Ethics

The study was approved by the Gabonese Ministry of Health (GMH) and the National Ethics Committee. All data obtained are part of routine activities in sentinel sites that are under the administrative supervision of the Gabonese Ministry of Health (GMH). Prompt malaria diagnosis and accurate treatment, drug resistance monitoring and interventions coverage in sentinel sites are the main strategies for malaria control of the GMH represented by the MNCP. The Department of Parasitology-Mycology (DPM) is the reference laboratory for malaria survey including diagnosis, antimalarial drug resistance evaluation, treatment efficacy, and impact of control strategies. Women were informed about the study protocol, and their written consent was required prior to the interview and sample collection.

### 2.7. Statistical Analysis

All questionnaires were completed at the end of all interviews. Two data entry clerks double-entered the data using Epi info version 2000. All data were analyzed with Stata 9.2 (Stata Corporation, College Station, TX, USA). Qualitative variables are summarized as frequencies and percentages. The median and interquartile range (25th and 75th percentiles) were used for continuous variables, such as parasitemia. Chi-squared tests and Fisher's exact tests were used for the comparison of proportions. A *P* value of less than 0.05 was considered significant.

## 3. Results

Overall, 44 women were selected, more than half *n* = 25 (57%) were multiparous. The median age was 26 [23–34] years; nine were aged less than 20 years and 14 were between 20 and 24 years old. Primiparous women had a median age of 20 [18–24] years. All women reported sleeping under a mosquito net during the pregnancy. The frequency of IPTp-SP use was 33% (*n* = 8/24) in the group of women selected in 2005 and 85% (*n* = 17/20) in 2011 (Table 1).

Overall eleven samples were microscopically infected (Table 2); among the peripheral blood samples (*n* = 5), the median microscopic parasite density was 294 [45–801] p/*μ*L, while parasitaemia varied from 8 to 378 p/*μ*L with a median of 16 [11–152] p/*μ*L in placental blood (*n* = 6 samples).

The proportion of matched concordant negative samples (33/44) was higher than the proportion of discordant samples, whatever the study period considered (Table 1). The frequency of concordant matched samples was higher in 2005 (83%) compared to 2011 (65%), but this difference was not statistically significant (*P* = 0.16 Fisher's exact test; Table 1). Among discordant samples (*n* = 11), those with microscopy negative results (MN) in placental blood tended to be more frequent in 2011 than in 2005 (20% versus 4% in 2005), whereas the proportion of microscopy positive (MP) placental blood samples was similar in both years (Table 1).

*P. falciparum* DNA was amplified in all samples shown to be infected by microscopy examination (*n* = 11); one concordant negative pair of matched samples also tested negative by PCR. Submicroscopic* Plasmodium* infections were detected in most (41/44) of the MN samples of peripheral (90% *n* = 35/39) and placental (87% *n* = 33/38) blood. The prevalence of submicroscopic infections did not differ significantly as a function of the level of IPTp-SP coverage: 95% in 2011 versus 79% in 2005 (Table 3) (*P* = 0.27).

All infected placental blood samples were matched with peripheral blood samples displaying microscopic or submicroscopic infection (Table 3). The frequency of concordant true-negative matched samples was less than 10% (*n* = 3/44; 7%). Only one of the pairs of discordant samples (peripheral positive/placental negative) was discordant for both microscopy and PCR (Table 4).

Microscopy failed to detect the malaria parasite in most of the samples of placental and peripheral blood collected from women with a HFTA (Table 3).

## 4. Discussion

The epidemiological profile of malaria is changing in Gabon, so additional information is required for the development of effective control strategies [17]. Malaria in pregnancy is characterized by the accumulation of* P. falciparum*-infected erythrocytes in placental intervillous spaces. Thus, placental malaria infection can be detected only at delivery, frequently in the absence of peripheral blood infection [21]. Tools for detecting the parasite and estimating the true risk of placental malaria before delivery are limited. A history of fever treated with antimalarial drugs (HFTA) with or without biological diagnosis, and the molecular detection of the malaria parasite in cases of peripheral and placental malaria would be helpful in this respect. A HFTA is associated with a higher frequency of placental and peripheral malaria at delivery. It therefore constitutes a good indicator of the presence of the parasite in women giving birth [22, 23].

This study confirms that the molecular detection of* P. falciparum* provides valuable information about the burden of peripheral and placental malaria. Submicroscopic infection was detected in most of the microscopy negative samples (more than 80%), consistent with previous data from Gabon, and for pregnant women, non pregnant adults, and children from Mozambique, Tanzania, and Congo [10, 13, 14, 24, 25]. The observed discrepancies between the results obtained with the two methods probably reflect the inclusion criteria, particularly for a HFTA, which is a recognized risk factor for placental* Plasmodium *infection. However, the relationship between HFTA and submicroscopic infection was not well identified in our setting, in which antimalarials are frequently prescribed presumptively to pregnant women presenting fever at ANCs, and in an era in which IPTp-SP treatment is widespread.

An absence of peripheral blood infection is not predictive of an absence of parasite in the placenta. Indeed, all venous blood samples from women with positive PCR results for placental blood displayed microscopically undetectable parasitemia, confirming the strong relationship between gestational malaria and submicroscopic placental and/or peripheral parasitemia at delivery [22]. In a study performed in Colombia, 94% of women with no HFTA tested negative at delivery [23]. Thus, the persistence of peripheral parasitemia at delivery is a matter of great concern. Nurses, obstetricians, and pregnant women should be informed on its potential impact on birth weight and its contribution to maternal anemia and puerperal malaria. Pregnant women should be regularly screened for malaria during pregnancy at least with microscopy or rapid diagnostic tests and treated if required. Most women presenting fever during visits to ANCs received an antimalarial drug, mostly quinine, which, unlike ACTs, is not active against ring-stage parasites. ACTs have been shown to decrease cumulative parasite biomass and to prevent high levels of hemozoin deposition in the placenta [26]. Their use from the second trimester, a period with a high frequency of pregnancy associated malaria, should be encouraged.

Another surprising result is the lack of effect of IPTp-SP coverage on the frequency of submicroscopic infection. This finding may reflect the small size of the sample used for PCR analysis. However, most of the women included in 2011 received only two doses of IPTp-SP, which is now known not to have a significant impact on microscopic or submicroscopic* P. falciparum* parasitemia [15, 25]. Indeed, the administration of two doses of SP seems to be sufficient to decrease parasite density but not to achieve complete clearance, as reported elsewhere [15, 27]. The administration of too low dose of SP and the high frequency of molecular markers of SP resistance in circulating isolates may partly account for the observed residual parasitemia [15, 27, 28]. Treatment with SP generally leads to the development of resistance [6], and clearance of the resident parasite therefore takes longer than clearance of sensitive strains. This could lead to persistence of the parasite at low densities not detectable by conventional microscopy. Between 2005 and 2011, the frequency of* dhfr/dhps* multiple mutations in isolates from pregnant women increased significantly, with the proportion of isolates displaying such mutations reaching more than 85% (Bouyou-Akotet submitted). Gametocytemia in the absence of asexual forms and the persistence of nucleic acids after parasite clearance may also yield positive test results.

## 5. Conclusion

In summary, these data highlight the high frequency of submicroscopic peripheral and placental* P. falciparum* infection, probably due to residual uncleared gestational infections. Discordant results for peripheral and placental blood seem to be otherwise infrequent. There is a need for accurate diagnostic tools for regular antenatal screening, to promote the early detection and prompt treatment of malaria episodes during pregnancy.

## Figures and Tables

**Table 1:** Baseline characteristics of the delivering women.

	All		2005		2011	
	*N* = 44		*n* = 24		*n* = 20	
	*N*	%	*n*	%	*n*	%
No IPTp	21	48	18	**75**	3	15
IPTP	23	52	**6**	**25**	17	85
1 dose	8	35	6	33	2	10
2 doses	6	14	0	0	6	30
3 doses	9	20	0	0	9	45
MN peripheral	39	89	23	96	16	80
MN placental	38	86	21	88	17	85

MN: microscopy negative, MP: microscopy positive.

**Table 2:** Concordant and discordant blood sample pairs distribution according to microscopy results.

	Peripheral blood			
	Microscopy positive		Microscopy negative	
	*N*	%	*N*	%
Placental blood				
Microscopy positive	0	0	6	14
Microscopy negative	5	11	33	75

**Table 3:** Distribution of pair samples PCR results according to IPTp-SP coverage.

	All		Low IPTp coverage		High IPTp coverage		
	(*N* = 44)		(*n* = 24)		(*n* = 20)		*P*
	*N*	%	*n*	%	*n*	%	
Concordant negative	3	7	3	12	0	0	**0,30**
Concordant positive	38	86	19	79	19	95	**0,28**
Neg^*^ peripheral/pos^**^ placental	1	2	1	4	0	0	NA^***^
Pos peripheral/neg placental	2	4	1	4	1	5	NA

^*^negative; ^**^positive; ^***^not applicable.

**Table 4:** PCR results by compartment.

PCR results	MN peripheral/MN placental			MN peripheral/MP placental			MP peripheral/MN placental		
	All	2005	2011	All	2005	2011	All	2005	2011
Neg peripheral/neg placental	3	3	0	0	0	0	0	0	0
Neg peripheral/pos placental	1	1	0	0	0	0	0	0	0
Pos peripheral/neg placental	1	1	0	0	0	0	1	0	1
Pos peripheral/pos placental	28	15	13	6	3	3	4	1	3

Total pairs	33	20	13	6	3	3	5	1	4

Neg: PCR negative; Pos: PCR positive; MN: microscopy negative; MP: microscopy positive.
